# A Woman with Vaginal Bleeding After Starting a New Oral Contraceptive

**DOI:** 10.1016/j.acepjo.2025.100323

**Published:** 2026-01-21

**Authors:** Emily Hartley Grant, Brooks Davis, Lindsay Tjiattas-Saleski

**Affiliations:** 1 Prisma Health, Greenville, Greer, South Carolina; 2 Edward Via College of Osteopathic Medicine Carolinas, Spartanburg, South Carolina

## Patient Presentation

**Figure 1:**
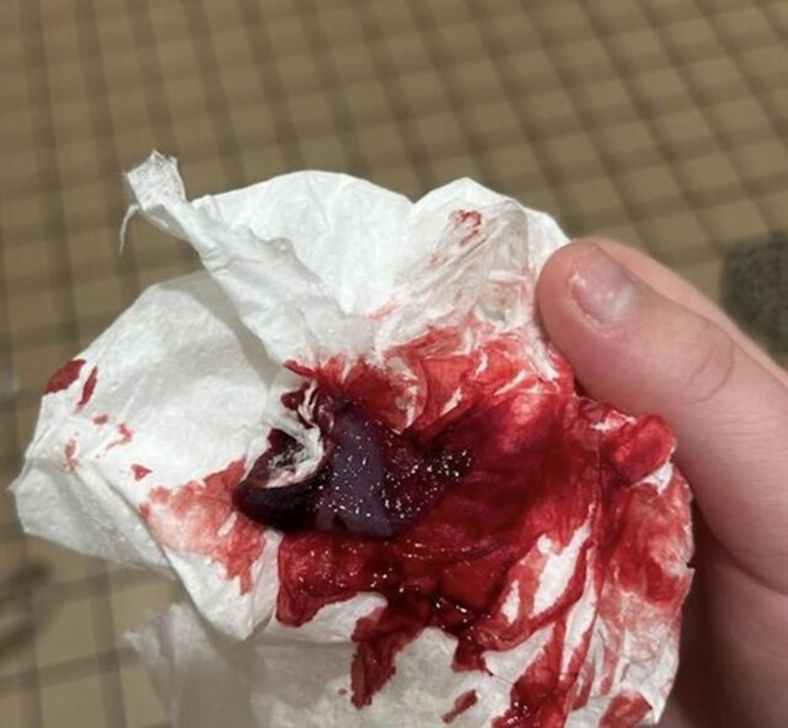
Tissue shed by patient, depicting a decidual cast.

A 19-year-old female presented to the emergency department with vaginal bleeding for 1 day and passage of what she believed to be fetal tissue due to possible miscarriage (Fig. 1). She endorsed initial abdominal cramping and lower back discomfort. She reported being sexually active and recent placement on a combined oral contraceptive (OCP), levonorgestrel and ethinyl estradiol (Simpesse), 2 months prior to presentation. She denied menses since starting the OCP. Her vitals were stable; abdominal and pelvic exams were unremarkable. Her urinalysis, complete blood count, and basic metabolic panel were unremarkable. Pelvic ultrasound showed a normal uterus and endometrium; the right adnexa was poorly visualized, which made it difficult to rule out an ectopic from imaging alone. Quantitative human chorionic gonadotropin (hCG) was normal for nonpregnant premenopausal women, ruling out ectopic pregnancy.

## Diagnosis/Teaching Points

OBGYN was consulted, who advised that given negative quantitative hCG and an unremarkable pelvic ultrasound, the patient likely shed a decidual cast. A decidual cast, also known as membranous dysmenorrhea, describes the shedding of the intact uterine lining that retains the form of the uterus.^1^^,^^2^ Decidualization of the endometrium, primarily mediated by progesterone, occurs after ovulation in preparation for potential embryonic implantation.^3^^,^^4^ Shedding of a decidual cast has been noted in patients taking oral contraceptives with a progestin component.^2^ Patients can present with abdominal pain and vaginal bleeding. They may have recently started a combined oral contraceptive therapy.^4^ Although rare, decidual casts are a differential diagnosis in female patients of reproductive age with vaginal bleeding with unremarkable labs and imaging.

## Funding and Support

By *JACEP Open* policy, all authors are required to disclose any and all commercial, financial, and other relationships in any way related to the subject of this article as per ICMJE conflict of interest guidelines (see www.icmje.org). The authors have stated that no such relationships exist.

## Conflict of Interest

All authors have affirmed they have no conflicts of interest to declare.
